# Management and Prevention of Pre-Eclampsia in Nigeria

**DOI:** 10.3390/healthcare11131832

**Published:** 2023-06-23

**Authors:** Oluwabunmi Victoria Adeyeye, Nwikwu Vivian Ebubechukwu, Omotayo Faith Olanrewaju, Aderayo Grace Eniayewun, Chidinma Nwuta, Fortune Benjamin Effiong, Brigid Unim

**Affiliations:** 1 Directorate of Research, TORASIF, Calabar 540004, Nigeria; bvcadeyeye@gmail.com (O.V.A.); ebubevivian24@gmail.com (N.V.E.); omotayofaith0@gmail.com (O.F.O.); aderayo.eniayewun@gmail.com (A.G.E.); chinwuta@gmail.com (C.N.); effiongfortuneb@gmail.com (F.B.E.); 2 Department of Medicine and Surgery, University of Ibadan, Ibadan 200005, Nigeria; 3 Faculty of Pharmaceutical Sciences, University of Nigeria Nsukka, Nsukka 410001, Nigeria; 4 Faculty of Medical Laboratory Science, University of Calabar, Calabar 540004, Nigeria; 5 African Community for Systematic Reviews and Meta-Analyses (ACSRM), Kigali 4285, Rwanda; 6 Department of Cardiovascular, Endocrine-Metabolic Diseases and Aging, Istituto Superiore di Sanità, Via Giano della Bella 34, 00162 Rome, Italy

**Keywords:** pre-eclampsia, burden, management, prevention, national policies

## Abstract

This research paper analyses the management and prevention of pre-eclampsia in Nigeria. Although efforts have been made to reduce outcomes due to pre-eclampsia, it still rears its head in the form of high maternal and perinatal morbidity and mortality. The aim of this review was to identify the main obstacles, gaps, and interventions related to the prevention and management of pre-eclampsia in order to be fully knowledgeable of the magnitude of the issue at the national level, to assess if current government policies are adequate and to recommend solutions. A search was performed on online databases and it was completed with hand searches related to the subject matter. Screening tests for early detection of pre-eclampsia are hardly available in Nigeria as many hospitals rely on the history of previous and current pregnancies, blood pressure monitoring and urinalysis–proteinuria. The administration of low-dose aspirin, antihypertensive drugs and magnesium sulphate, coupled with calcium in calcium deficit regions, was recommended. The main barriers to the wider implementation of these strategies are inadequacy of the antenatal care services in providing appropriate care, lack of resources and trained personnel, high healthcare costs, and low antennal care attendance. Improving education and awareness, use of low-cost screening modalities and low-dose aspirin can be deployed in developing countries to curb pre-eclampsia.

## 1. Introduction

Pre-eclampsia is a condition characterized by hypertension and significant proteinuria, usually occurring after 20 weeks of gestation in a woman that was previously normotensive and non-proteinuric. The blood pressure value indicative of hypertension is >140/90 mmHg and that of significant proteinuria is >0.3 g/day or a urine protein/creatinine ratio >30 mg/mmol [1]. Women who have a family and previous history of pre-eclampsia, diabetes mellitus, obesity, or thrombosis, and those whose index pregnancy is their first are at a higher risk of pre-eclampsia [2]. The incidence of pre-eclampsia varies widely; it is seven times higher in developing countries compared with industrialized countries [2], ranging from 1.8% to 16.7% [3,4,5,6]. In Nigeria, the prevalence ranges between 2% to 16.7%, which is similar to other developing nations [7,8,9].

Early detection of pre-eclampsia is important to prevent complications [1], such as eclampsia, kidney or liver damage, and the death of the mother and/or foetus [1,10]. The treatment of pre-eclampsia involves the administration of magnesium sulphate at varying doses [1,10]; aspirin is also utilized in the management of pre-eclampsia [11]. However, magnesium sulphate remains the drug of choice [1,10]. Statins have been proved beneficial in women with antiphospholipid syndrome, pre-eclampsia or foetal growth restriction, by improving uteroplacental perfusion [12].

This article aims at understanding the main challenges and interventions related to the prevention and management of pre-eclampsia in Nigeria. In this light, literature sources were searched on online databases such as PubMed, Google Scholar, and ResearchGate, and hand searches related to the subject matter were performed. Keywords such as *pre-eclampsia, management, prevention,* and *national policies* were applied.

## 2. Burden of Pre-Eclampsia

According to the World Health Organization (WHO) estimates, about 300,000 maternal deaths occurred in 2017 worldwide, of which over 60% were in Sub-Saharan Africa. Nigeria (67,000), India (35,000), Democratic Republic of Congo (16,000), Ethiopia (14,000), and Tanzania (11,000) were the five countries with the highest number of maternal deaths in 2017. Postpartum haemorrhage, pre-eclampsia, sepsis, unsafe abortions and obstructed labour are the most frequent direct causes [13]. 

Pre-eclampsia and eclampsia-related issues are considered the cause of between 50,000 to 75,000 deaths in women each year [13]. The United Nations Millennium Development Goals (MDGs) 4 and 5 aimed at reducing child mortality by two-thirds and maternal mortality by three quarters by 2015 [14]. Furthermore, the preventable maternal mortality (EPMM) statement released by the WHO seeks to reduce the global maternal mortality ratio to less than 70 per 100,000 live births by 2030 [2]. These goals are directly addressed by improving health care services associated to pre-eclampsia.

## 3. Pathophysiology of Pre-Eclampsia

**Figure 1:**
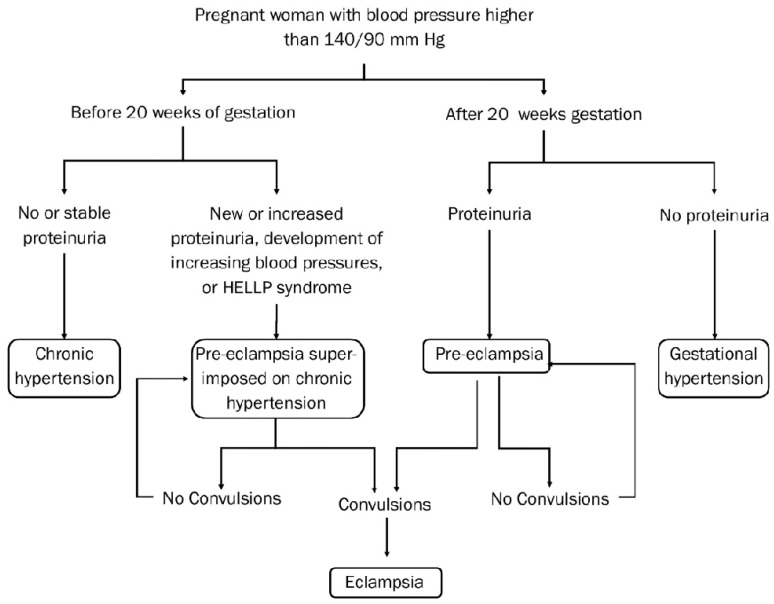
Flowchart of pre-eclampsia (Adapted from [10]. (HELLP syndrome: haemolysis, elevated liver enzymes, low platelet count).

Pre-eclampsia is a condition where a woman experiences a rapid elevation of blood pressure to >140/90 mmHg (hypertension) and high levels of protein in the urine (significant proteinuria >0.3 g/day or urine protein/creatinine ratio >30 mg/mmol) after 20 weeks of gestation. If untreated, it can progress to eclampsia, a leading cause of maternal and perinatal mortality globally (Figure 1) [1,10]. Pre-eclampsia is mostly caused by poorly developed uterine placental spiral arteries. Normally, the uterine placental arteries undergo physiological transformation, a remodelling process, and dilate up to 10 times their normal size to allow increased blood flow between the mother and foetus [15]. In the case of pre-eclampsia, the uterine placental spiral arteries are fibrous, causing them to narrow, with a reduced blood flow to the placenta [1]. The altered blood flow causes ischemia-reperfusion injury (intervals of hypoxia followed by reoxygenation of the placenta) and placental oxidative stress [16]. This leads to the release of inflammatory placental factors into the maternal circulation, thus to maternal inflammatory response to the released factors, resulting in generalized vascular inflammation. This process lays the basis for the clinical manifestation of pre-eclampsia [17,18]. 

Pre-eclampsia can also be caused by poor placentation that increases the risk of placental dysfunction. In other words, extrinsic factors (e.g., diabetes, multiple pregnancies, or hypoxic condition of the mother due to anaemia) could alter the placental barrier with the release of apoptotic fragments and this may lead to maternal immune response, resulting in the clinical symptoms of pre-eclampsia. The same may occur if the maternal disposal or inflammatory systems are not functioning properly and react inappropriately to the release of apoptotic fragments, thus inducing a systemic immune response that results in pre-eclampsia [18].

## 4. Signs and Symptoms of Pre-Eclampsia

Pre-eclampsia may be asymptomatic or may cause excessive weight gain or oedema of the face and hands because of increased vascular permeability and proteinuria. In the case of severe pre-eclampsia, the signs and symptoms are visual impairment; severe headaches, confusion (caused by cerebral oedema); nausea/ vomiting; dyspnoea (due to pulmonary oedema, acute respiratory distress syndrome, or cardiac dysfunction); epigastric pain (caused by hepatic ischemia or capsular distention); impaired liver function; and oliguria (related to the decreased plasma volume). Petechiae or other signs of coagulopathy may develop [1,10]. Pre-eclampsia is also characterized by increased neuromuscular irritability, related to magnesium deficiency, and may progress to seizures (eclampsia) [19].

## 5. Complications of Pre-Eclampsia

Women affected by severe pre-eclampsia or eclampsia have a higher risk of HELLP syndrome (Hemolysis, Elevated liver enzymes, and Low Platelets). It is a rare syndrome with similar pathogenesis with pre-eclampsia, and occurs in 0.1% to 0.8% of all pregnancies [20], but the incidence increases to 20% in women with pre-eclampsia [21] and 27.6% in those with eclampsia [22]. It should be noted that hypertension and proteinuria are not always present in women with HELLP syndrome [23]. The incidence of HELLP syndrome varies globally and most women with HELLP are multiparous, older (over 35) and of white ethnicity [24]. In the United States, 15% of pregnant women with pre-eclampsia develop HELLP (about 45,000 women/year) [25]. In Thailand, the incidence rate of pre-eclampsia and HELLP found in a recent study was 9.5 per 1000 deliveries [26]. Regarding African countries, including Nigeria, a systematic review found a pool prevalence of HELLP syndrome of 2.2 per 1000 pregnancies. The review also highlighted that about half of the diagnosed cases in Africa are severe pre-eclampsia, which is much higher compared to developed countries. For instance, in the United States, severe pre-eclampsia accounts for 25% of all pre-eclampsia cases [27].

Pre-eclampsia patients are also at risk of placental abruption in their current and future pregnancies, as both disorders are related to uteroplacental insufficiency. Placental abruption increases the risk of maternal, foetal or neonatal morbidity and mortality [1]. Other possible complications of pre-eclampsia are foetal growth restriction, a placental syndrome characterized by poor remodelling of the spiral arteries, and intrauterine foetal death [1]. The association of pre-eclampsia with higher risk of maternal outcomes, such as cardiovascular disease in Canadian and Norwegian populations, heart failure in Canadian and Danish populations, stroke in Denmark and Scotland, and hypertension in the UK and Denmark, is reported in the literature [28].

## 6. Detection and Prevention of Pre-Eclampsia

Effective treatment of pre-eclampsia depends on early detection that can be achieved by early identification of the risk factors such as a family history of pre-eclampsia, antiphospholipid syndrome, nulliparity, obesity, increased maternal age, chronic hypertension, kidney disease, diabetes, multiple pregnancy, etc. There have been attempts to develop an effective mode of screening for pre-eclampsia to enable early detection. In these approaches, a combination of maternal serum biomarkers in early pregnancy (i.e., pregnancy-related plasma Protein-A, Inhibin-A, and placental growth factor) are considered an accurate screening model for early onset pre-eclampsia in nullipara [29]. 

These screening tests are hardly available in Nigeria, as many hospitals still rely on history of previous and current pregnancies, blood pressure monitoring and urinalysis–proteinuria [29]. Other factors related to early detection and prevention of pre-eclampsia in Nigeria are antenatal care (ANC) attendance and the quality of the ANC services. Although the WHO recommends at least eight ANC contacts during pregnancy, only 20% of pregnant women met this recommendation in Nigeria in 2021 [30]. Delays in care seeking are common, as most women are in their second or third trimester at the time of their first ANC booking [31]. Inadequate maternal-care-seeking behaviour has been associated with low educational level, low income, high cost of health care services, and cultural factors such as the influence of traditional birth attendants and low decision-making power [30]. ANC attendance is also related to difficulties in accessing health facilities due to distance and transportation difficulties, especially in rural areas [24]. Furthermore, inadequacies of the ANC services in providing appropriate and correct care have been documented in some facilities (e.g., lack of equipment such as an ultra sound scanner, non-routine administration of magnesium sulphate, lack of optimally trained personnel, and referral delays to other medical practitioners) [31]. 

Regarding prophylaxis, the effectiveness of aspirin in cases of high risk of pre-eclampsia without former hypertensive complaint of gravidity is not certain yet. Aspirin is effective in secondary prevention of pre-eclampsia mainly in cases with a history of pre-eclampsia. In primary prevention of pre-eclampsia, low-dose aspirin is given in the first trimester by carrying out netting tests. This seems to reduce the occurrence of pre-eclampsia at an early stage [11]. Pregnant women at moderate/high risk of pre-eclampsia or with chronic hypertension should be given 75–150 mg of aspirin daily from 12 weeks until delivery [32,33]. 

Calcium supplementation is also recommended in women with low calcium intake (<600–900 mg/day) while anti-hypertensives should be administered to women with severe hypertension [32,34,35]. There is evidence that an optimal concentration of calcium and other electrolytes (i.e., magnesium) can stabilize vascular cell membranes, inhibit calcium entry into cells, and reduce vasoconstriction, thus reducing blood pressure. The concentrations of these electrolytes tend to decrease during pregnancy due to physiological changes and major reductions occur in women with pre-eclampsia [36]. The recommended daily intake of supplements during pregnancy is 1500–2000 mg/daily for calcium [34] and 300 mg for magnesium [37]. 

The use of aspirin as a preventive strategy is not widely adopted in Nigeria, and calcium supplementation is also offered as prophylaxis in few states [35]. Although the Nigerian Federal Ministry of Health has included all necessary drugs for the management of hypertensive disorders during pregnancy (Labetalol, Hydralazine, Methyldopa, Nifedipine, magnesium sulphate, and calcium gluconate) on the national Essential Medicines List and recommended their regular supply in each health facility, these medications are not routinely available in most facilities [10,35]. The implementation of these recommendations may be limited by resource constraints (drug/supplement availability, lack of trained staff), and the associated cost. 

## 7. Management and treatment of Pre-Eclampsia 

The main treatment for pre-eclampsia is prompt delivery, mostly after maternal stabilization (e.g., blood pressure, seizures). Pre-eclampsia and eclampsia usually resolve within 6 to 12 h after delivery. If delivery can be delayed (e.g., pregnancies of less than 34 weeks), corticosteroids are given for 48 h to enhance maturity of the foetal lung. Magnesium sulphate is recommended in patients with severe pre-eclampsia or eclampsia to prevent seizures and should be continued for 12 to 24 h after delivery. Magnesium sulphate 4 g in 20 min is administered intravenously, followed by an intravenous infusion of 2 g/h. Women with high magnesium levels after receiving magnesium sulphate can be treated with a 1 g intravenous load of calcium gluconate [1,32,38].

In Nigeria, treatment of pre-eclampsia has relied on antenatal use of aspirin, a vasodilator (high doses of aspirin dilate blood vessels through direct effect on vascular smooth muscle), and anti-hypertensives like calcium channel blockers safe for pregnancy with various adoption rates across the country [35]. Delivery is planned as soon as possible, and intrapartum management employs the use of magnesium sulphate administered using varying clinical protocols. Two common dosing rules of magnesium sulphate are available and are named after two doctors, Pritchard and Zuspan. The Prichard protocol consists of a loading cure of 14 g (intravenous administration of 4 g and intramuscular injection of 10 g), followed by a 5 g intramuscular injection every 4 h for 24 h. The Zuspan protocol is an intravenous loading cure of 4 g, followed by intravenous infusions of 1 g per hour for 24 h [39]. Contraindications to intravenous magnesium sulphate include myasthenia gravis or other neuromuscular diseases, severe renal failure, cardiac ischemia, heart block, diabetic coma, and pulmonary edema [40]. Given that infusion pumps are generally not available in most developing countries, the Pritchard protocol is preferred in low-income countries; however, it is associated with pain and a higher risk of infection at the injection site [41]. Calcium gluconate is used as an antidote for magnesium sulphate toxicity [10,39].

Although magnesium sulphate is recommended at the national level, the drug is often not available at primary care levels or may not be administered according to guidelines. A research and capacity building project was conducted in the period of 2007–2017 to improve availability and correct use of magnesium sulphate in Nigerian hospitals. A reduction of 40% in maternal mortality was achieved in Kano State (Northern Nigeria). The project also enabled the provision of magnesium sulphate in 40 primary health care facilities and the development of a national training programme on the management of pre-eclampsia and eclampsia with magnesium sulphate [9].

## 8. Recommendations

Globally, pre-eclampsia affects 4.6% of pregnancies. Research studies have shown a greater prevalence of pre-eclampsia, up to 16.7% in Nigeria [7,8,9]. This high prevalence is linked to a significant percentage of unbooked women who do not obtain ANC services from trained healthcare providers before presentation in labour. The maternal and prenatal health in Nigeria can be addressed by taking steps to prevent and mitigate the morbidity and mortality caused by pre-eclampsia. Risk evaluation, screening, and diligent clinical management must be ensured in clinical settings. This is achievable through routine history taking, assessment of risk factors, and clinical investigations, including blood pressure measurement and urinalysis.

According to the WHO, ‘strengthening women’s and community engagement in maternal health promotes a positive experience for all involved, further strengthening a country’s health system and improves access to high quality, respectful maternal health care for every woman’ [42]. It has been demonstrated that the bulk of intrapartum maternal mortality occurs in poorly/underperforming health systems. Efforts must be made to strengthen the Nigerian healthcare system through the mobilization of sufficient resources for health, availability of trained personnel, medication, and equipment in order to increase the quality of obstetric services, especially in rural areas where an optimal referral system must be developed [29]. The WHO recommends that each country’s health workers should review existing guidelines to include clear practical guidelines for community health workers and authorized skilled birth attendants to give magnesium sulphate and anti-hypertensives in cases of severe pre-eclampsia and eclampsia. Trained healthcare workers can also provide calcium in areas where there is calcium deficiency. Moreover, a screening checklist should be developed for pregnant women with risk factors [43]. Barriers to the implementation of these recommendations include resource availability (i.e., staff and supplies), cultural beliefs and perceptions of healthcare services, knowledge and awareness of the condition and available interventions.

Health promotion strategies at the community level about risk factors, symptoms, prevention, and potential consequences of pre-eclampsia, are necessary to create awareness and actions to reduce maternal morbidity and mortality due to pre-eclampsia. It is important to raise awareness on the importance of prompt ANC attendance and home-based blood pressure monitoring in the detection of hypertension in pregnancy [44]. This could help in increasing the likelihood of therapeutic interventions and reducing complications [45,46]. Lifestyle modifications such as rest, physical activity, reduction in salt intake could be discussed at this level. Although daily rest may reduce the risk of pre-eclampsia for women with normal blood pressure, the evidence is insufficient to recommend rest as a preventive strategy. Likewise, physical activity in women at risk of pre-eclampsia cannot be recommended due to insufficient evidence [47]. Reducing dietary salt is a valid practice for the general population and hypertensive patients; however, current guidelines do not recommend reduced salt intake solely to prevent pre-eclampsia [33,38]. Priority should be given to strategies that have a broader impact and are feasible in Nigeria, such as the use of low-dose aspirin, public preventive campaigns and training programmes addressing healthcare providers, starting from basic skills.

A limitation of the review is the paucity of recent published records about the management and prevention of pre-eclampsia in low- and middle-income countries, including Nigeria. Most studies or reports have been published prior to 2008. Likewise for data regarding these settings cited in international reports or guidelines. Future studies with more recent data will enable a more accurate description and comparisons within and across countries.

In conclusion, a strong political agenda is essential in reducing the burden of maternal mortality and morbidity associated with pre-eclampsia. Adequate healthcare funding, improving education and empowerment in the society are strategies that can be deployed by the government in curbing the debilitating effect of pre-eclampsia [48]. Further clinical research should also be carried out to ascertain the exact pathophysiology of pre-eclampsia, and accurate and inexpensive screening modalities should be devised to enhance the prevention of pre-eclampsia, especially in low- and middle-income countries.

## Data Availability

The data presented in this study are available on request from the corresponding author.
